# Clinical Ethics Support Services Are Not as Well-Established in Forensic Psychiatry as in General Psychiatry

**DOI:** 10.3389/fpsyt.2020.00186

**Published:** 2020-03-13

**Authors:** Irina Franke, Oskar Speiser, Manuela Dudeck, Judith Streb

**Affiliations:** ^1^Department of Forensic Psychiatry and Psychotherapy, Ulm University, Ulm, Germany; ^2^Psychiatric Services Graubuenden, Department of Forensic Psychiatry, Cazis, Switzerland

**Keywords:** clinical ethics support, clinical ethics, ethics consultation, forensic mental health, forensic psychotherapy

## Abstract

**Background:** Mental health care professionals deal with complex ethical dilemmas that involve the principles of autonomy, justice, beneficence, and non-maleficence. Such dilemmas are even more prominent in forensic mental health care, where the restriction of personal rights is legitimated not only by patient well-being but also by public safety interests. Little is known about either the use of formal ethics support services or specific ethical needs in forensic mental health care. Knowledge about the current structures and how they compare with those in general psychiatry would help to identify the most important ethical issues and to analyze whether there are unmet needs that might require specific ethics support.

**Methods:** We performed a survey study in all general psychiatric and forensic psychiatric inpatient departments in Germany. The aims were to compare the availability and functioning of clinical ethics structures and to identify specific ethical needs in inpatient forensic and general mental health care.

**Results:** Clinical ethics support was available in 74% of general psychiatric hospitals but in only 43% of all forensic psychiatric hospitals and 25% of those offering treatment for offenders with substance use disorders. Most ethics support services were interdisciplinary. The most frequently requested retrospective and prospective ethics consultations were on issues of omission and termination of treatment, coercive measures, and advance directives. Among the hospitals without access to ethics support, 71% indicated a need for training in ethics.

**Discussion:** Our results show that ethics consultation is well established in general psychiatry, but less so in forensic psychiatry. Mental health care professionals in forensic psychiatry seem to have a need for ethics support and training in clinical ethics. We also found a difference in access to ethics structures between hospitals that treat mentally disordered offenders and those that treat offenders with substance use disorders. Further research should focus on how ethics support can be comprehensively implemented in forensic mental health care and how this might improve treatment quality and patient and staff well-being.

## Introduction

In the past two decades, the four normative principles of clinical ethics put forward by Beauchamp and Childress (1) have become the most important guideline for ethical decision making in health care. According to those principles, all health care professionals have a duty to promote patient autonomy, avoid harm (non-maleficence), do what is best for the patient (beneficence), and respect applicable laws and people's rights and distribute resources fairly (justice). Every therapeutic decision—including to terminate treatment—is supposed to be based on the patient's wishes and informed consent. Although Beauchamp and Childress stated that the four principles do not follow a hierarchical order and that none of them should be seen as a normative absolutism (1), the question whether, for example, the patient's right to decide autonomously outweighs the right to physical and mental integrity in the context of coercive treatment interventions is still a matter of discussion, especially in psychiatry. The underlying ethical dilemmas tend to be even more complex in forensic mental health care, where the infliction of harm by third parties or the adverse effects of substandard living conditions have to be taken into account (2). Ethical decision making in clinical practice is challenging because mental health professionals are rarely given training in how to apply ethical guidelines in individual cases. Moreover, most of the ethics standards in general psychiatry and psychology [for example (3, 4)] do not cover the specific ethical conflicts that arise in forensic psychiatry and psychotherapy.

In several other fields of medicine, clinical ethics support (CES) has become a valuable and effective tool for solving such ethical dilemmas and reflecting therapeutic decisions. CES-teams offer ethics consultations, training programs, and guidelines that are supposed to provide guidance for clinicians on decision making in complex clinical situations. Ethics consultation is defined as “a service provided by an individual consultant, team, or committee to address the ethical issues involved in a specific clinical case. Its central purpose is to improve the process and outcomes of patients' care by helping to identify, analyze, and resolve ethical problems” (5). Such consultations can be requested by professionals who are involved in a patient's treatment and by the patient and relatives. The decision-making process does not aim to come to a majority decision, but to find a consensus that can be accepted by all involved persons. It should further consider the legal framework of treatment and current scientific standards. CES teams should offer regular meetings and education for patients, relatives, and the general public to improve the awareness of and competence in dealing with ethical issues. Special curricula have been developed to train clinical ethics consultants in the required competences [see, for example, (6)]. In Germany, the number of hospitals offering clinical ethics consultation has steadily increased in the last two decades (7). In psychiatry, however, structured ethics consultation has developed at a slower rate than in other medical disciplines, but it is considered to be relevant and helpful for moral case deliberation and an unprejudiced decision-making process (8–10). A study on clinical ethics consultations in Norway showed that 144 of 775 cases between 2003 and 2012 related to mental health and addiction treatment cases; among the most prominent ethical dilemmas were confidentiality and information (33 cases), drug dependency (27 cases), formal and informal coercion (23 cases), and competence to consent and patient autonomy (16 cases) (11).

Ethical case reflection has to take into account the legal framework of treatment decisions, especially concerning preconditions for admission and coercive measures. In Germany, admission to forensic psychiatric inpatient treatment is based on Sections 63 and 64 of the criminal code. Detention according to Section 63 requires diminished or no criminal responsibility resulting from a diagnosis of a severe mental disorder (e.g., schizophrenia, intellectual disability, severe personality disorder). In contrast to several other European countries, in Germany the duration of detention is not limited by law; however, the longer the detention lasts, the more relevant considerations of proportionality, i.e., the risk of severe re-offending against the right to freedom, become (12). Patients detained according to Section 64 have a substance use disorder (often with comorbid personality disorder), which rarely affects criminal responsibility. Detention according to Section 64 has a legally defined time limit of two years. Additionally, both the therapist and the detainee can request a court decision to terminate treatment according to Section 64 if there are no longer any realistic prospects of successful treatment. With respect to the different legal backgrounds, patients detained according to Section 63 clearly have a lot in common with general psychiatric patients and their treatment causes similar ethical conflicts, especially regarding the use of coercion. After a 2011 high court decision in which forced antipsychotic medication was declared to be a severe encroachment on the right to physical integrity, legislation had to be revised in several federal states. Subsequently, coercive medication practices became more restrictive. In the meantime, there is some evidence that this change not only led to an increase in the use of physical restraint and seclusion, but also to a deterioration of the atmosphere on wards and more violent conflicts in forensic-psychiatric hospitals (13).

Even though in its 2017 consensus statement on standards in forensic mental health care in Germany an interdisciplinary expert group emphasized that the four normative principles autonomy, non-maleficence, beneficence, and justice must be the basis of any therapeutic decision (14), little is known about clinical ethics and the role of CES in forensic mental health care in Germany. Gather et al. (15) found that in the federal state of North Rhine-Westphalia, only 29% of all forensic psychiatric hospitals had some kind of CES, whereas 90% of general psychiatric hospitals offered such services.

This survey study aimed to provide insight into the current state of CES in German forensic and general psychiatric inpatient treatment. To do so, it examined the availability, organizational structures, resources, institutional implementation, and prominent ethical conflicts in these two kinds of hospitals. Furthermore, as part of the study hospitals without current ethics structures were asked to express their needs for CES. The results might help to increase awareness for ethical conflicts and decision-making processes in forensic psychiatry.

## Materials and Methods

### Procedures

We used a modified questionnaire originally developed for the assessment of clinical ethics structures in Switzerland (16–18). The main modifications were the addition of a section to assess the needs of hospitals without an established CES structure, the revision of items that mentioned specific national laws or legal terms, and the integration of features characteristic of forensic psychiatric treatment. The modified questionnaire was imported into the survey software Unipark® (QuestBack GmbH Cologne, Germany) and e-mailed to the medical directors or representatives of the 240 general psychiatric and 70 forensic psychiatric hospitals in Germany, as listed in the published registers for each district. If we were unable to find an e-mail address for a hospital, we contacted it by telephone and asked for the medical director's contact details or requested that the questionnaire be forwarded to the medical director. Because we received only 30 responses within 6 weeks, we decided to prepare a paper-pencil version of the online questionnaire, which we then sent, together with a personal cover letter, to the directors of the hospitals that had not yet responded. If we received duplicate responses (i.e., both the online and paper-pencil questionnaires), we included the questionnaire with the fewest missing responses in our analyses.

### Sample

We contacted 310 general psychiatric and forensic psychiatric hospitals by e-mail and regular mail, and 119 questionnaires were returned. Of these, three had to be excluded from further analyses because the questionnaire had been completed by outpatient treatment services. An additional five responses had to be excluded because we received both an online and a paper-pencil response. Thus, a total of 111 questionnaires (85 paper-pencil, 26 online) were available for analysis, corresponding to a response rate of 36%. The sample included 65 general psychiatric hospitals, 30 forensic psychiatric hospitals, and 16 hospitals that provide both general psychiatric and forensic psychiatric treatment. The 16 hospitals providing both forms of care were excluded from the comparative analyses between forensic and general psychiatry. The analysis of response rates according to the states in which the hospitals were located approximately matched their relative population size and indicated a representative distribution of responses. Only for Bavaria, Hesse, and Mecklenburg-Western Pomerania were the response rates slightly higher than the states' relative population size. The majority of responses were obtained from North Rhine-Westphalia (20%), Bavaria (19%), Lower Saxony (13%), Baden-Wuerttemberg (12%), and Hesse (10%).

### Assessments

All participating hospitals were asked to provide general information, e.g., bed capacity, operator, and special treatment focus. Depending on whether CES was available or not, they were then asked to complete either version A (for hospitals with CES) or version B (for those without CES). Descriptions of CES were provided on the questionnaire.

Version A comprised 29 items referring to organizational structures of CES; competence and responsibility; year of implementation; frequency of specific ethical problems/conflicts; additional unmet needs; institutional integration, including financial and human resources. To assess the issues handled by CES, we used a 4-point Likert scale ranging from 1 (= never) and 4 (= very often). In addition, we assessed the size and professional backgrounds of the CES team; whether training and supervision were available and mandatory; the number of meetings/consultations per year; the processing time from request to recommendation; how the consultations were documented; and public perception of the CES.

Version B comprised 25 items that estimated the frequency of potential ethical problems, potential responsibilities of a CES team, preferred professional competences if CES were to be implemented, preferred time for processing a request, and training needs. As for Version A, hospitals were asked to rate these items on a 4-point Likert scale ranging from 1 (= never) and 4 (= very often).

### Statistical Analyses

Statistical analyses were performed with IBM SPSS Statistics Version 25 (2017). For categorical variables, relative frequencies were compared with the Pearson χ^2^-test. If more than 20% of the expected cell frequencies were smaller than five, the Fisher's exact test was calculated instead. Because none of the distributions met the criteria for a parametric *t-*test, responses on the 4-point Likert scales were compared with the Mann–Whitney *U*-Test. To measure effect sizes, we used Cohen's *d* for mean differences and Cramer's *V* for frequencies. Significant results were tested two sided, with an α-level of 5%. As mentioned above, the hospitals (*n* = 16) that provided both general psychiatric and forensic psychiatric treatment were excluded from the comparative analyses between forensic and general psychiatry.

## Results

### Availability, Organization, and Integration of CES

#### Availability of CES

In total, 73 hospitals (66%) confirmed having a CES structure. The prevalence of CES was higher in general psychiatric hospitals (74%) and hospitals providing both general psychiatric and forensic psychiatric care (75%) than in forensic psychiatric hospitals (43%) [χ^2^_(2)_ = 9.196, *p* = 0.010, Cramer's *V* = 0.288, see Table 1].

**Table 1:** Absolute and relative frequencies of clinical ethics support (CES) according to type of hospital.

**Type of hospital**	**CES available**		**Total**
	**Yes**	**No**	
	**Absolute (relative) frequency**	**Absolute (relative) frequency**	**Absolute (relative) frequency**
General psychiatry	48 (74%)	17 (26%)	65 (100%)
Forensic psychiatry	13 (43%)	17 (57%)	30 (100%)
General and forensic psychiatry	12 (75%)	4 (25%)	16 (100%)
Total	73 (66%)	38 (34%)	111 (100%)

Furthermore, we found that 83% of the forensic psychiatric hospitals that offered treatment according to Section 63 of the German criminal code (severe mental disorder) provided CES, but only 25% of those offering treatment according to Section 64 (substance use disorders). Hospitals that provided both forms of treatment provided CES in 40% (see Table 2).

**Table 2:** Absolute and relative frequencies of clinical ethics support (CES) in forensic psychiatric hospitals according to the type of treatment provided (Section 63 of the German criminal code: involuntary treatment for severe mental disorders; Section 64: involuntary treatment for substance use disorders).

**Type of treatment provided**	**CES available**		**Total**
	**Yes**	**No**	
	**Absolute (relative) frequency**	**Absolute (relative) frequency**	**Absolute (relative) frequency**
According to Section 63	15 (83%)	3 (17%)	18 (100%)
According to Section 64	2 (25%)	6 (75%)	8 (100%)
According to Section 63 and 64	8 (40%)	12 (60%)	20 (100%)
Total	25 (54%)	21 (46%)	46 (100%)

Overall, the bed capacity (<100, 101–300, and >300 beds) had no influence on the availability of CES [$\chi_{\left(2\right)}^{2}$ = 4.718, *p* = 0.108, Cramer's *V* = 0.206]. However, non-profit institutions and university hospitals were more likely to provide CES than public or private institutions [$\chi_{\left(3\right)}^{2}$ = 8.084, *p* = 0.044, Cramer's *V* = 0.270].

#### Organization of CES

The most prevalent organizational form of CES was the clinical ethics committee (78%). Among the 50 hospitals that provided the year when the CES structure was implemented, the majority (76.5%) indicated 2008 or later. CES teams consisted of a mean (*SD*) of 10.5 (3.47) members and met 6 (3.47) times per year. They handled an average of 10 (18.08) cases per year and could be consulted within a period of 12 h (from request to meeting) in 30% of the institutions. In 38% of the institutions, urgent issues could be handled within 24 h. No statistically significant differences were found between general psychiatric and forensic psychiatric hospitals (Fisher's exact test = 5.569, *p* = 0.232, Cramer's *V* = 0.290).

The most prevalent professions in CES teams (valid *n* = 71) were physicians (100% of cases), nursing staff (94%), and pastoral care staff (90%). External experts were employed by 72% of the institutions. In 38% of CES teams, members were required to complete subject-specific ethics training. Peer supervision was not offered or required by 76% of the CES teams. Compared with CES teams in general psychiatric hospitals, CES teams in forensic psychiatric hospitals more often included social workers [χ^2^_(1)_ = 11.103, *p* = 0.001, Cramer's *V* = 0.434].

Hospitals without CES indicated that they would want to have physicians (100%), nursing staff (87%), professional ethicists (82%), and legal experts (76%) represented in CES teams. Additionally, they demanded more expertise in intercultural competence and philosophy than is available in existing CES teams (see Table 3). Participating representatives in forensic psychiatric hospitals without CES indicated a need for nursing staff in CES teams less often than those in general psychiatric hospitals without CES [χ^2^_(1)_ = 5.862, *p* = 0.044, Cramer's *V* = 0.415].

**Table 3:** Professions represented on or requested in clinical ethics support (CES) teams.

	**Hospitals with CES**	**Hospitals without CES**
	**Frequency (%)**	**Frequency (%)**
Medicine	100	100
Nursing	94	87
Spiritual welfare	90	68
Ethics	48	82
Law	41	76
Psychology	48	53
Social work	47	34
Administration	44	24
Intercultural competence	4	45
Philosophy	7	21

#### Integration of CES

We assessed the institutional integration of CES structures by asking about formal regulations and available resources. Overall, 97% of the CES structures had regulations defining their responsibility and functioning and 82% received financial resources; personnel resources were provided in 64% of the institutions. The majority of participating institutions with CES allowed active CES members (71%) to participate in ethics training respectively ethics consultation (78%) during working hours; however, staff were allowed to do so in only 37% of these hospitals (see Table 4). We found no significant differences between forensic and general psychiatric hospitals. Among the participating hospitals with CES, 26% declared that they had too few resources to provide clinical ethics training to staff, whereas this was the case in 71% of the institutions without CES. We found no significant differences between general psychiatric and forensic psychiatric hospitals.

**Table 4:** Institutional integration of clinical ethics support (CES) structures.

	**Frequency (%)**
Regulations clarify the assignment and operating principles	97
The institution provides financial resources (e.g., for further training in ethics)	82
Members may engage in ethics consultation during working hours	78
Ethics consultation is well known and accessible in the institution	75
Members may engage in ethics training during working hours	71
The institution provides personnel resources (e.g., administration or office support)	64
Ethics consultation is closely connected to the medical director	57
The institution's staff may engage in ethics consultation during working hours	37

We also asked participants to indicate whether and how CES teams actively communicated with staff, patients, and relatives. Most CES structures disseminated information on ethics consultations via internal publications for staff (69%). Clinical ethics training (39%), the personal address of department directors (38%), and invitations to meetings (e.g., ethics cafés; 24%) were available less often. Only 22% of CES teams actively approached patients and their relatives, and 22% did not actively disseminate any information. There was no significant difference between general psychiatric and forensic psychiatric institutions.

### Requests and Cases

#### Hospitals With CES

The majority of the requests for support from CES teams involved individual prospective (71%) and retrospective (81%) ethics consultations. Additional requests concerned general ethical decision making (53%), developing clinical guidelines (58%), providing training in ethics (53%), and counseling for hospital directors (48%). Only a few CES teams also engaged in issues related to clinical research (4%). We found no significant differences between general psychiatric and forensic psychiatric hospitals; however, we did find differences between institutions of different sizes and types. Institutions with <100 beds requested training in ethics less often than larger institutions [χ^2^_(2)_ = 6.359, *p* = 0.046, Cramer's *V* = 0.295, see Table 5], and non-profit institutions requested advice in general ethical decision making more often than public, private, or university hospitals [χ^2^_(3)_ = 10.791, *p* = 0.012, Cramer's *V* = 0.384]. Compared with public, private, or non-profit institutions, university hospitals asked less often that clinical guidelines be developed [χ^2^_(3)_ = 10.141, *p* = 0.016, Cramer's *V* = 0.373] and sought advice concerning research in the field of clinical ethics more often [χ^2^_(3)_ = 7.637, *p* = 0.045, Cramer's *V* = 0.323, see Table 6].

**Table 5:** Absolute and relative frequencies of types of clinical ethics support requested according to the size of the institution.

	**Bed count**		
	**<100**	**101–300**	**>301**
	**Absolute (relative) frequency**	**Absolute (relative) frequency**	**Absolute (relative) frequency**
Prospective ethics consultation	12 (71%)	27 (71%)	13 (72%)
Retrospective ethics consultation	15 (88%)	30 (79%)	14 (78%)
General ethical decision making	7 (41%)	21 (55%)	11 (61%)
Developing clinical guidelines	7 (41%)	23 (61%)	12 (67%)
Providing training in ethics	5 (29%)	25 (66%)	9 (50%)
Counseling for hospital directors	8 (47%)	19 (50%)	8 (44%)
Clinical research	0	1 (3%)	2 (11%)

**Table 6:** Absolute and relative frequencies of types of clinical ethics support requested according to the type of the institution.

	**Organizational type of hospital**			
	**Public**	**Private**	**Non-profit**	**University**
	**Absolute (relative) frequency**	**Absolute (relative) frequency**	**Absolute (relative) frequency**	**Absolute (relative) frequency**
Prospective ethics consultation	31 (84%)	8 (62%)	7 (64%)	6 (50%)
Retrospective ethics consultation	33 (89%)	10 (77%)	8 (73%)	8 (67%)
General ethical decision making	18 (49%)	8 (62%)	10 (91%)	3 (25%)
Development of clinical guidelines	24 (65%)	8 (62%)	8 (73%)	2 (17%)
Providing training in ethics	20 (54%)	8 (62%)	8 (73%)	3 (25%)
Counseling for hospital directors	20 (54%)	5 (39%)	8 (73%)	2 (17%)
Clinical research	0	0	1 (9%)	2 (17%)

The ethical conflicts most frequently dealt with related to termination of treatment procedures (*M* [*SD*] = 2.52 [0.92]), advance directives (*M* [*SD*] = 2.44 [0.86]), coercive measures (medication: *M* [*SD*] = 2.35 [0.84]; physical restraint (*M* [*SD*] = 2.18 [0.85]); seclusion (*M* [*SD*] = 1.81 [0.79]); and [attempted] suicide (*M* [*SD*] = 2.03 [0.93]); the issues least frequently dealt with were non-indicated interventions (*M* = 1.50 [0.71]), data protection (*M* [*SD*] = 1.48 [0.64]), diagnosis evaluation (*M* [*SD*] = 1.46 [0.64]), and pregnancy termination (*M* [*SD*] = 1.40 [0.61]). In forensic psychiatry, participants indicated significantly more cases regarding seclusion (*Z* = −2.189, *p* = 0.029, Cohen's *d* = 0.631) and professional/lawful conduct (*Z* = −2.060, *p* = 0.039, Cohen's *d* = 0.573) than in general psychiatry (see Table 7).

**Table 7:** Mean (SD) frequency of specific ethical issues dealt with by clinical ethics support (CES) teams (1 = never; 2 = rarely; 3 = often).

	**General psychiatry**	**Forensic psychiatry**
	***M* (*SD*)**	***M* (*SD*)**
Coercive medication	2.28 (0.84)	2.67 (0.89)
Advance directives	2.36 (0.91)	2.50 (0.90)
Physical restraint	2.11 (0.81)	2.50 (1.00)
Treatment discontinuation	2.64 (0.91)	2.36 (1.21)
Lawful and professional behavior	1.84 (0.80)	2.36 (0.67)
Seclusion	1.71 (0.71)	2.36 (0.92)
Conflicting values within team	2.04 (0.93)	2.18 (0.98)
Artificial nutrition	2.23 (0.84)	2.09 (1.14)
Conflicts with patients' relatives	2.00 (0.88)	2.09 (0.83)
Conflicts between patients and staff	1.80 (0.84)	2.00 (0.77)
Suicide and attempted suicide	2.09 (1.03)	1.91 (0.67)
Emergencies	2.05 (0.87)	1.91 (0.90)
Confidentiality	1.62 (0.72)	1.92 (0.67)
Intercultural issues	1.74 (0.66)	1.90 (0.99)
Data protection	1.48 (0.69)	1.72 (0.47)
Risk assessment	1.98 (0.95)	1.70 (0.82)
Dealing with cognitively challenged patients	1.88 (0.91)	1.70 (0.48)
Research with patients and their data	1.61 (1.05)	1.70 (0.95)
Conflicts between staff	1.73 (0.81)	1.58 (0.79)
Indication for surgery	1.58 (0.76)	1.55 (0.69)
Economic interests	1.56 (0.70)	1.50 (0.85)
Wish-fulfilling medicine	1.57 (0.77)	1.50 (0.71)
Diagnostic assessment	1.44 (0.66)	1.40 (0.52)
Pregnancy discontinuation	1.45 (0.67)	1.25 (0.46)

We also asked participants to indicate the estimated frequency of consultation requests according to the professional background of the person making the request; responses were provided on a 4-point Likert scale ranging from 1 (= not yet) to 4 (= very often). CES was requested most often by nursing management (*M* [*SD*] = 2.34 [0.96]), nursing staff (*M* [*SD*] = 2.33 [0.93]), and chief or head physicians/medical directors (*M* [*SD*] = 2.16 [0.96]). Requests from patients (*M* [*SD*] = 1.84 [0.78]) and their relatives (*M* [*SD*] = 1.79 [0.82]) were considerably rarer. Compared with general psychiatric hospitals, in forensic psychiatric hospitals CES teams were approached more often by hospital directors (*Z* = −2.470, *p* = 0.014, Cohen's *d* = 0.706) and heads of departments (*Z* = −2.062, *p* = 0.039, Cohen's *d* = 0.568).

#### Hospitals Without CES

Participating institutions without CES were asked to estimate for which ethical conflicts they would potentially require CES. These hospitals indicated needs for retrospective ethics consultation (82%), individual ethical decision making (72%), prospective ethics consultation (60%), development of clinical ethics guidelines (50%), counseling advice for the medical director (47%), training in clinical ethics (34%), and ethics advice for clinical research (18%). Compared with the institutions with CES, the indicated need was significantly higher for retrospective ethics consultation (*Z* = −2.67, *p* = 0.012, Cohen's *d* = 0.524), counseling advice for the medical director (*Z* = −2.74, *p* = 0.010, Cohen's *d* = 0.539), and development of clinical ethics guidelines (*Z* = −3.36, *p* = 0.001, Cohen's *d* = 0.673).

Concerning specific ethical conflicts, general (non–case-specific) value conflicts within the care team (*M* [*SD*] = 2.26 [0.76]), conflicts between physicians, therapists, and nursing professionals (M [*SD*] = 2.19 [0.71]), conflicts between the care team and patients or relatives (*M* [*SD*] = 2.11 [0.52]), risk assessment (*M* [*SD*] = 1.91 [0.71]), professional and lawful conduct (*M* [*SD*] = 2.08 [0.64]), intercultural competence (*M* [*SD*] = 2.16 [0.69]), and clinical research on patients (*M* [*SD*] = 1.91 [0.71]) were indicated as the main needs. General psychiatric hospitals stated having a significantly higher need for CES regarding physical restraint (*Z* = −2.376, *p* = 0.018, Cohen's *d* = 0.926), advance directives (*Z* = −2.688, *p* = 0.007, Cohen's *d* = 1.080), and pregnancy termination (*Z* = −3.598, *p* < 0.001, Cohen's *d* = 1.743). Forensic psychiatric hospitals without CES required ethics support regarding clinical research more often than general psychiatric hospitals (*Z* = −2.438, *p* = 0.015, Cohen's *d* = 0.994).

## Discussion

Various forms of CES have been established over the last two decades to promote ethical reflection and professional ethical conduct in health care and—with a certain delay—in mental health care. With this study, we aimed to examine and compare CES structures in general psychiatric and forensic psychiatric hospitals in Germany. To find out whether there are substantial differences in the availability, responsibility, and requirements of CES, we performed a nationwide interview study.

Although two thirds of the participating general and forensic-psychiatric hospitals declared that they had access to CES, one of our main findings was a noticeable difference in the availability of CES between forensic and general psychiatry, i.e., forensic psychiatric hospitals reported having less access to CES than general psychiatric hospitals. However, the rate of 43% found in this survey for the whole of Germany is slightly higher than the rate of 29% found by Gather et al. (15) for the district of North Rhine-Westphalia, which might indicate that clinical ethics is developing but still not well established in forensic psychiatry. Moreover, we found a substantial difference in the availability of CES between forensic psychiatric hospitals that offer treatment according to Section 63 (83%) and those treating patients according to Section 64 (25%). The low rate of CES in the latter types of hospitals was surprising because we assumed that although these hospitals might have different ethical conflicts, they would not have fewer. This discrepancy might be explained by the characteristics of the two populations (severe mental disorder vs. substance use disorder, often with comorbid personality disorder) and differences in the treatment itself (duration, termination). Thus, patients detained according to Section 63 have a lot in common with general psychiatric patients, resulting in comparable ethical conflicts, especially regarding the use of coercive measures. In contrast, patients detained according to Section 64 rather display features of prison populations than of psychiatric inpatients; in these settings, implicit coercion might be more prevalent than explicit coercion, meaning that ethical conflicts may be overlooked. Another reason for the discrepancy might be the misconception that patients detained according to Section 64 are not a vulnerable group or do not require the same standards of ethical conduct as patients with severe mental disorders. Unfortunately, because of the relatively low response rate of hospitals providing treatment according to Section 64 we were unable to analyze their subjective clinical ethics needs separately.

The overall availability of CES did not depend on the size of the hospital, but on the organizational structure, i.e., non-profit and university hospitals were more likely to have access to CES. Possible explanations for this finding might be the longer history of clinical ethics and specific organizational value systems in some types of hospital, for instance church-operated ones, and the distribution of financial resources within the organization. The most prevalent organizational form of CES was the clinical ethics committee with multidisciplinary members, mainly with medical backgrounds. Surprisingly, specific training for the team members was required in less than 40% of the clinical ethics teams and regular peer supervision was rather rare. Over two thirds of the clinical ethics teams were able to offer consultations within 24 h, which was surprising because most of the ethical conflicts in psychiatry are not as acute as in somatic medicine.

Regarding the organizational structure and institutional implementation of CES, we found no significant differences between general psychiatric and forensic psychiatric hospitals. Remarkably, only 37% of the participating hospitals allowed staff to take part in ethics consultations during working hours, which might have a negative effect on accessibility and acceptance of ethical case consultation in teams. CES in forensic psychiatric hospitals seems to be structured in a slightly more hierarchical way and focused on the profession of medicine. The majority of CES requests in forensic-psychiatric hospitals were made by the medical director's office. In accordance with this observation, forensic psychiatric hospitals without CES expressed less need for nursing professionals in ethics consultation teams.

In general, requests for ethics consultation were heterogeneous and no specific issues were significantly prominent. In both general and forensic psychiatry, the most frequent requests concerned coercive medication, advance directives, discontinuation of treatment, and physical restraint. The high frequency of requests regarding coercive medication and physical restraint might be interpreted as a consequence of the restrictive laws in Germany concerning forced medication in forensic and general psychiatry. According to the literature, however, coercive measures in general are among the most prominent moral conflicts handled in psychiatric ethics consultations (8, 11, 19). Requests for ethics consultation on seclusion and ethical/lawful conduct were more common in forensic psychiatry. The above mentioned legal context might explain why ethics consultation is often requested for seclusion: Patients tend to be secluded for a significantly longer time because they can neither be treated on the ward without posing a risk to themselves or others nor receive medication against their will (13). The higher number of requests regarding ethical/lawful conduct probably reflects an increased awareness of the restriction of autonomy or individual rights of patients in long-term treatment settings. It might also reflect moral and professional uncertainty among staff as to how to act correctly within the given legal, ethical, and professional boundaries in forensic psychiatric treatment. We additionally found that the focus of ethics consultations differs with respect to the size and organization of the hospital: Smaller hospitals requested clinical ethics training less often, non-profit institutions asked for advice on general ethical decision making more often, and university hospitals sought counseling regarding research more often.

Hospitals that did not yet have access to ethics structures expressed a need for professional diversity, e.g., the involvement of intercultural competence and philosophy. This might be interpreted in the context of an increasing number of patients with a migration background, especially among people in detention in forensic psychiatry according to Section 64 (20). Furthermore, hospitals without available CES structures more often indicated that they would require ethics support in moral value conflicts between team members, between people from different professions, and with relatives. This finding underlines the important role of CES in moral case deliberation in mental health care (8). Further support for the benefit and effectiveness of CES comes from the finding that only 26% of hospitals with active CES requested further resources for training in clinical ethics, whereas 71% of the hospitals without access to CES did.

Even though our results support the view of CES as a helpful instrument for professionals and teams, one should note that patients and their relatives are currently largely uninvolved in CES. Even if information about CES is distributed to the public, inpatients or relatives seem to rarely be able to address the ethics committee themselves. Opening up CES for requests from patients and their relatives, or involving them more actively in case consultations, might support their autonomy, promote recovery and improve decision-making processes.

Although we obtained a broad range of information on CES in German forensic psychiatric institutions, our study has several limitations. First, although the overall response rate of 36% corresponds with those of comparable studies (7), it cannot be considered as being representative. Ethics might not play a significant role in non-responding hospitals; consequently, they would not participate in a survey assessing ethics support. Thus, a sample selection bias cannot be excluded. Because we obtained a significantly higher rate of responses (66%) from the forensic-psychiatric hospitals, the representativeness of the data regarding this sub-sample can be considered to be slightly better. Furthermore, switching from the e-mail to the paper-pencil version of the questionnaire meant that we were no longer able to ensure that participants answered the survey completely. As a consequence, we received paper questionnaires with missing data. Additionally, we were not able to examine in more detail hospitals treating offenders according to Section 64 of the criminal code. Because of the low availability of CES in this field of forensic psychiatry, it would be of further interest to explore potential institutional or personal obstacles to its implementation. Last, our results can only provide a first overview on ethics in forensic psychiatry. We did not specifically ask about methodological issues of CES or the handling of cases in detail (such as ethical reasoning for or against specific treatment measures).

In conclusion, in Germany CES is well established in general mental health care, but not yet in forensic-psychiatry, especially in the treatment of offenders with substance use disorders. Existing CES structures in forensic psychiatry do not differ from those in general psychiatry regarding organization, resources, and implementation. CES structures in both types of hospitals seem to cover a variety of ethical issues, with an emphasis on conflicts between patient autonomy and treatment decisions (i.e., coercive measures). Members of hospitals without CES clearly expressed a need for training in clinical ethics. CES seems to be a valid instrument for discussing ethical conflicts and promoting professional conduct in a challenging environment. However, patients and relatives are not yet very well integrated in the CES process. Further research should focus on evaluating CES structures in (forensic) mental health care.

## Data Availability Statement

The datasets generated for this study are available on request to the corresponding author.

## Author Contributions

IF, JS, and MD designed the study. OS collected the data. OS and JS analyzed the data. IF, OS, and JS interpreted the data. IF and OS wrote the initial draft of the manuscript. All authors had full access to all the data in the study and take responsibility for the integrity and accuracy of the data analysis. All authors contributed to, read and approved the final version of the manuscript.

### Conflict of Interest

The authors declare that the research was conducted in the absence of any commercial or financial relationships that could be construed as a potential conflict of interest.
